# Case Report: Primary epithelioid angiosarcoma of the femur: two rare cases with atypical skeletal involvement

**DOI:** 10.3389/fmed.2026.1955241

**Published:** 2026-10-02

**Authors:** Fangfang Xu, Min Xu, Qian Li, Fei Dong, Chao Wang

**Affiliations:** 1 Department of Radiology, The Second Affiliated Hospital, Zhejiang University School of Medicine, Hangzhou, China; 2 Department of Radiology, Jiande Branch, The Second Affiliated Hospital, Zhejiang University School of Medicine, Jiande, China

**Keywords:** case report, computed tomography, femur, immunohistochemical, magnetic resonance imaging, malignant vascular tumor, positron emission tomography/computed tomography, primary epithelioid angiosarcoma

## Abstract

**Background:**

Primary epithelioid angiosarcoma (EA) is an exceptionally rare malignant vascular tumor with aggressive behavior and nonspecific imaging features. Femoral involvement is particularly uncommon and may mimic other destructive bone neoplasms.

**Case report:**

We describe two men with primary EA of the femur. One patient presented with progressive right hip pain and a pathological fracture of the proximal femur, whereas the other presented with progressively worsening pain in the distal left thigh. Imaging demonstrated destructive osteolytic lesions with intramedullary involvement and variable extension into the adjacent soft tissues. Both patients underwent wide tumor resection followed by prosthetic reconstruction. Histopathological examination revealed malignant epithelioid vascular neoplasms, and immunohistochemical (IHC) positivity for endothelial markers supported the diagnosis.

**Conclusion:**

Primary femoral EA should be considered in the differential diagnosis of aggressive osteolytic lesions, particularly when relatively limited enhancement, hemorrhagic components, and adjacent soft tissue extension are present. Definitive diagnosis requires integrated assessment of radiological, histopathological, and IHC findings.

## Introduction

Epithelioid angiosarcoma (EA) is a rare, high-grade histologic variant of angiosarcoma arising from vascular or lymphatic endothelial cells and is characterized by aggressive clinical behavior and a poor prognosis (1–3). Angiosarcoma most commonly affects the skin and superficial soft tissues, whereas primary osseous EA is exceptionally rare (1–3). Its clinical manifestations are nonspecific and typically include localized pain, swelling, restricted joint mobility, and, occasionally, pathological fracture (3, 4).

Radiography and computed tomography (CT) of osseous EA typically reveal ill-defined osteolytic lesions with cortical destruction and possible extraosseous extension, whereas magnetic resonance imaging (MRI) is valuable for delineating intramedullary tumor extent and adjacent soft-tissue involvement (5, 6). However, these imaging features are nonspecific and may overlap with those of metastatic carcinoma, plasmacytoma, osteosarcoma, and other aggressive skeletal neoplasms, making an accurate preoperative diagnosis challenging (5, 6). Histologically, EA is composed predominantly of malignant epithelioid endothelial cells arranged in solid sheets, nests, or irregular vasoformative channels. Additionally, immunohistochemical (IHC) confirmation of endothelial differentiation, particularly through the expression of markers such as CD31 and ERG, is essential for establishing the diagnosis (2, 3, 6).

Herein, we report two rare cases of primary femoral EA that were initially interpreted as malignant bone tumors on imaging and subsequently confirmed by postoperative histopathologic and immunohistochemical (IHC) examination. These cases provide additional insight into the radiologic and pathologic characteristics of this rare entity and may improve clinical awareness, facilitate early recognition and accurate diagnosis.

## Case report 1

A 62-year-old man presented with progressive right hip pain that had persisted for one month without an identifiable precipitating event. The pain worsened during weight-bearing and was accompanied by mild local tenderness, restricted hip motion, and intermittent radiation to the right lumbar region and right knee. His past medical history was unremarkable. Physical examination revealed no additional remarkable abnormalities. Laboratory testing showed mildly elevated carbohydrate antigen 125 (51.3 U/mL), neuron-specific enolase (26.9 ng/mL), and C-reactive protein (90.9 mg/L). Notably, before admission to our institution, the patient underwent CT-guided needle biopsy of the right femoral lesion at another hospital. The biopsy was reported to show predominantly fibrotic and necrotic tissue, with insufficient viable tumor tissue for a definitive diagnosis; however, the original pathology report was unavailable for review. A preoperative diagnosis of plasmacytoma was initially considered.

Radiography demonstrated an expansile osteolytic lesion involving the right femoral neck, accompanied by cortical destruction and a pathological fracture (Figure 1A). CT further revealed extensive osteolytic destruction of the proximal femur and an intramedullary soft-tissue mass measuring approximately 64 × 59 × 92 mm, with an attenuation of approximately 37 Hounsfield units (HU) (Figures 1B,C). MRI showed a heterogeneous intramedullary lesion with predominantly hyperintense signal on T1-weighted imaging (T1WI) (Figure 1D). On T2-weighted imaging (T2WI), the lesion exhibited heterogeneous signal intensity with multiple internal hypointense foci, suggestive of hemosiderin deposition secondary to prior intratumoral hemorrhage (Figure 1E). Following contrast administration, the lesion showed mild heterogeneous enhancement, whereas the adjacent muscles and surrounding soft tissues demonstrated more conspicuous enhancement, indicating extraosseous extension (Figure 1F). The extraosseous component predominantly involved the anterior fascial compartment of the thigh, with extension into the iliopsoas and quadriceps femoris musculature, including the vastus lateralis. Positron emission tomography/computed tomography (PET/CT) revealed heterogeneous radiotracer uptake confined to the proximal right femur, with no evidence of additional skeletal lesions (Figure 1G).

**Figure 1:**
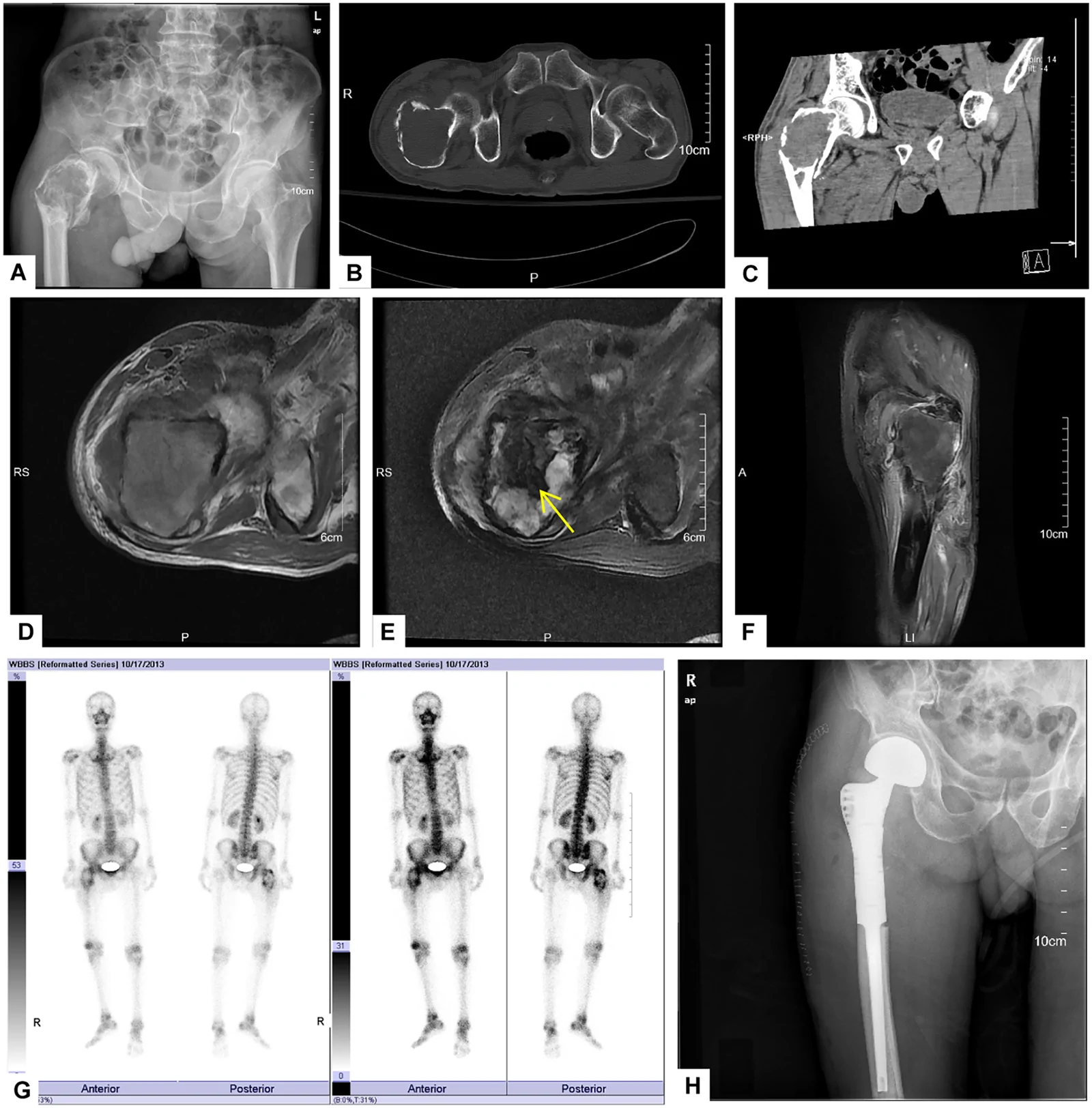
Imaging findings of the right proximal femoral lesion. **(A)** Radiograph demonstrated an expansile osteolytic lesion involving the right femoral neck, with cortical destruction and a pathological fracture. **(B)** Axial CT bone-window image demonstrated extensive osteolytic destruction of the proximal femur. **(C)** Coronal CT soft-tissue-window image showed an intramedullary soft-tissue mass. **(D)** Axial T1WI showed a heterogeneous lesion with predominantly hyperintense signal. **(E)** Axial T2WI demonstrated heterogeneous signal intensity with multiple internal hypointense foci, suggestive of hemosiderin deposition (arrow). **(F)** Contrast-enhanced T1WI showed mild heterogeneous enhancement of the lesion and more conspicuous enhancement of the adjacent muscles and soft tissues. **(G)** PET/CT demonstrated heterogeneous radiotracer uptake confined to the proximal right femurs. **(H)** Postoperative radiograph showed satisfactory alignment of the proximal femoral endoprosthesis.

Given the aggressive osteolytic destruction, pathologic fracture, intratumoral hemorrhage, and extraosseous soft-tissue extension, the initial differential diagnosis included metastatic bone disease, malignant giant cell tumor of bone, and solitary plasmacytoma of bone. After thorough discussion with the patient and his family and exclusion of relevant contraindications, the patient underwent wide resection of the proximal femoral lesion followed by endoprosthetic reconstruction. The procedure was completed uneventfully. Postoperative histopathological examination revealed infiltrative malignant epithelioid endothelial cells arranged in irregular anastomosing vascular channels and solid nests (Figures 2A,B), with abundant eosinophilic cytoplasm, nuclear pleomorphism, and evident mitotic activity (Figure 2C). IHC staining demonstrated that the tumor cells were positive for CD31, and ERG (Figures 2D,E). The Ki-67 proliferation index was approximately 20% (Figure 2F). These findings confirmed the diagnosis of primary EA of the femur. Postoperative radiography demonstrated satisfactory prosthetic alignment (Figure 1H). The patient subsequently returned to his local hospital for further postoperative treatment; however, details of the treatment regimen were unavailable. At the 3- and 6-month postoperative follow-up visits, no clinical evidence of local recurrence was observed, although follow-up imaging was unavailable for review.

**Figure 2:**
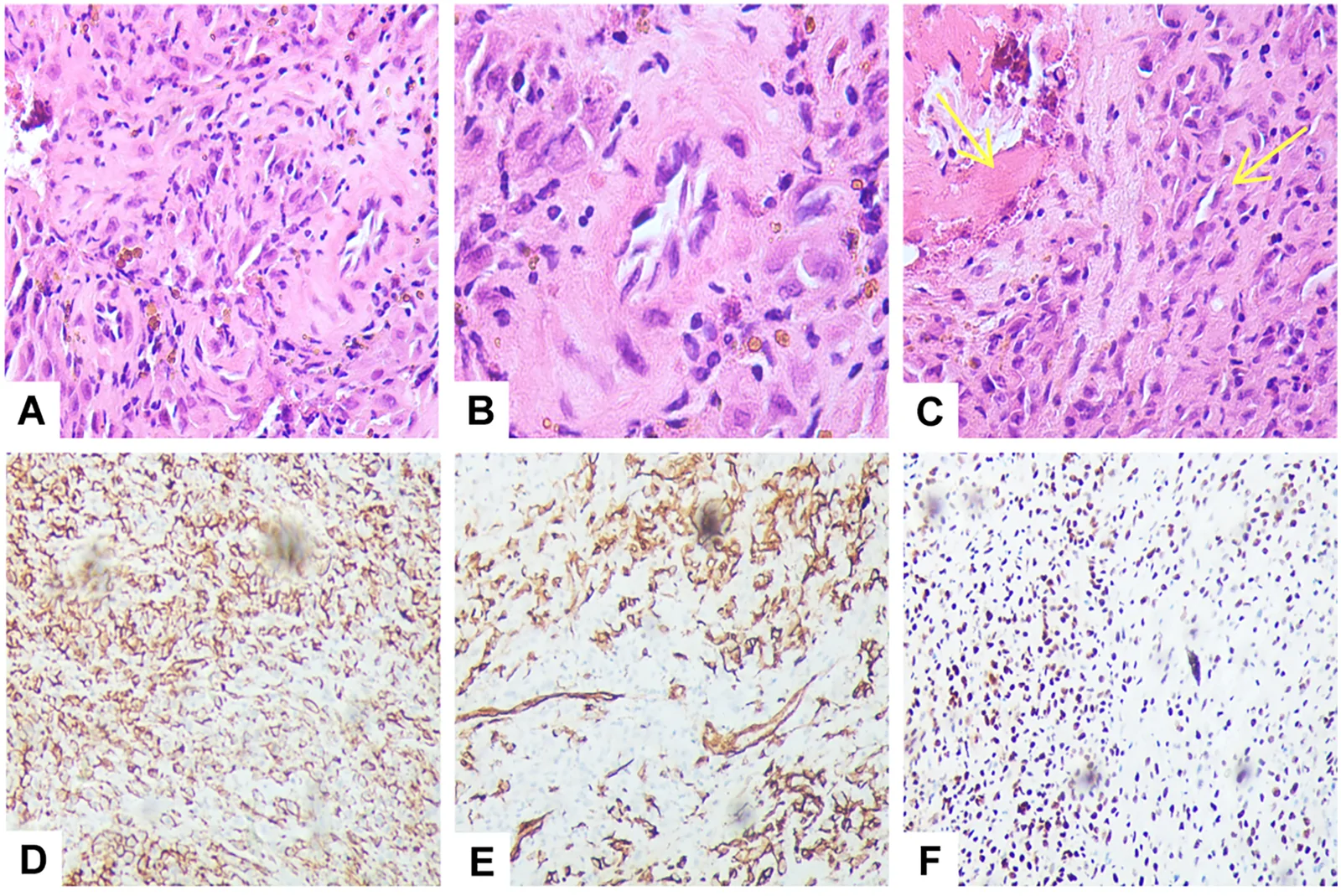
Histopathological and IHC findings of the right proximal femoral lesion. **(A,B)** Low- and high-magnification HE staining demonstrated infiltrative malignant epithelioid tumor cells lining irregular, anastomosing vascular channels and forming solid nests. **(C)** The tumor cells exhibited abundant eosinophilic cytoplasm, enlarged pleomorphic hyperchromatic nuclei, and readily identifiable mitotic figures. The left arrow indicates bone tissue, and the right arrow indicates tumor tissue. **(D)** CD31 was positive in tumor cell. **(E)** ERG was positive in tumor cell. **(F)** The Ki-67 index was approximately 20%.

## Case report 2

A 57-year-old man presented with a 15-day history of progressively worsening intermittent pain in the distal left thigh. The pain was described as paroxysmal and stabbing, without clear association with activity. Apart from a left femoral fracture 40 years earlier, his past medical history was unremarkable. Imaging at a local hospital suggested an intramedullary lesion in the distal left femur; however, the original images and radiological report were unavailable. He was therefore admitted with a presumptive diagnosis of a malignant femoral tumor.

Radiography revealed an expansile osteolytic lesion involving the distal femur without obvious cortical breakthrough (Figure 3A). CT demonstrated aggressive medullary destruction accompanied by intraosseous and extraosseous soft-tissue components containing residual osseous fragments; no periosteal reaction was observed (Figure 3B). Contrast-enhanced CT showed heterogeneous enhancement with internal linear septal enhancement (Figure 3C). MRI revealed a slightly hypointense intramedullary lesion on T1WI, with focal cortical destruction and an adjacent soft-tissue mass (Figure 3D). On T2WI, the lesion exhibited heterogeneous hyperintensity with internal septations and surrounding soft-tissue edema (Figure 3E). Contrast-enhanced T1WI showed mild heterogeneous enhancement of both the osseous lesion and adjacent soft-tissue component (Figure 3F). Fat-suppressed contrast-enhanced sagittal T1WI more clearly delineated the irregular enhancement, cortical disruption, and extraosseous extension of the lesion, which measured approximately 40 × 81 × 33 mm (Figure 3G). The extraosseous component predominantly involved the medial head of the gastrocnemius and was confined to the superficial posterior compartment of the leg. PET/CT demonstrated markedly increased heterogeneous radiotracer uptake within both the distal femoral lesion and its associated soft-tissue component, raising suspicion for a metabolically active malignant bone tumor (Figure 3H).

**Figure 3:**
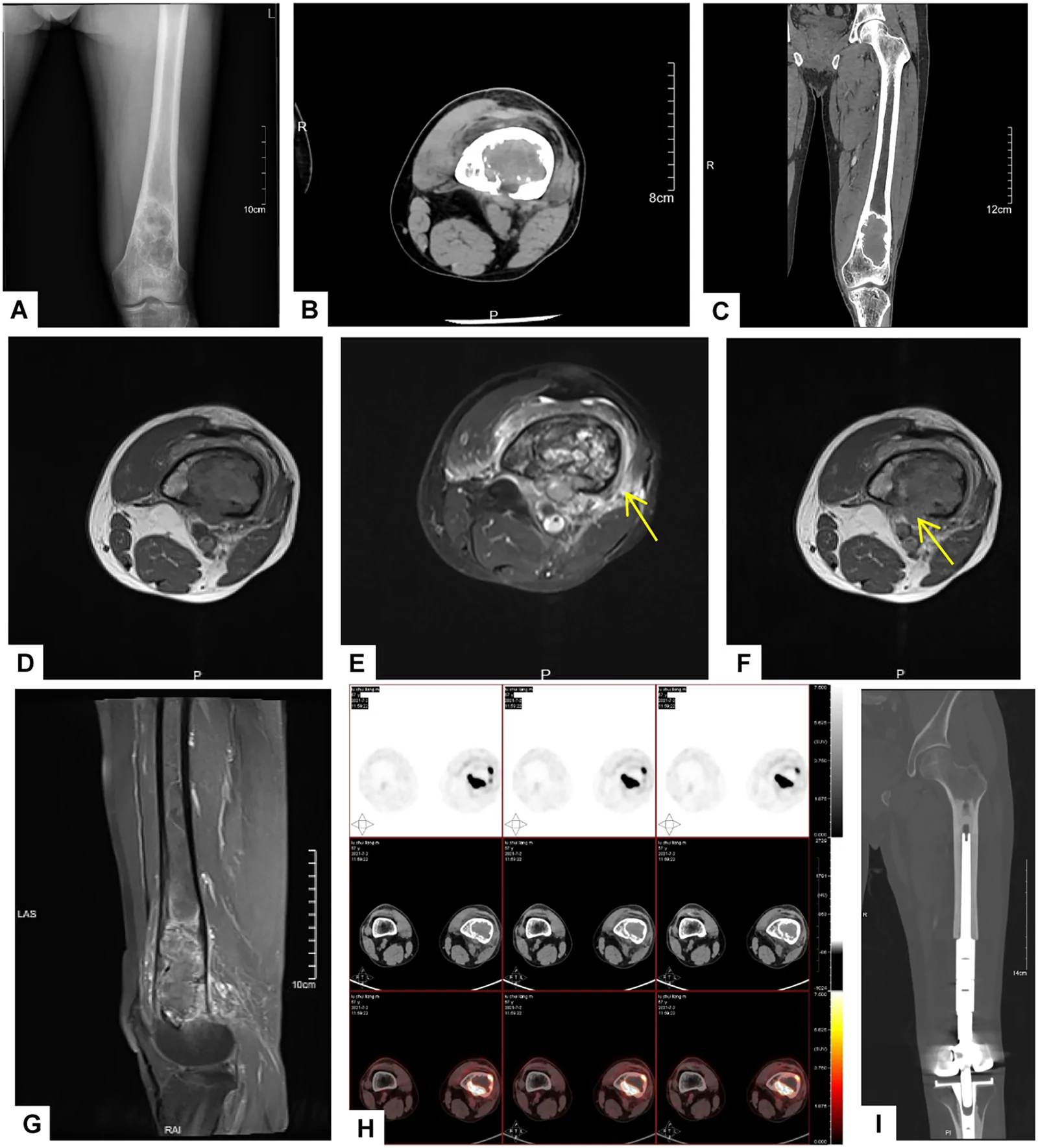
Imaging findings of the left distal femoral lesion. **(A)** Radiograph demonstrated an expansile osteolytic lesion involving the distal femur. **(B)** CT demonstrated aggressive medullary destruction with residual osseous fragments, intraosseous and extraosseous soft-tissue components. **(C)** Contrast-enhanced CT showed heterogeneous enhancement with internal linear septal enhancement. **(D)** Axial T1WI revealed a slightly hypointense intramedullary lesion with focal cortical destruction and an adjacent soft-tissue mass. **(E)** Axial T2WI demonstrated heterogeneous hyperintensity with internal septations and surrounding soft-tissue edema (arrow). **(F)** Axial contrast-enhanced T1WI showed mild heterogeneous enhancement of both the osseous lesion and the adjacent soft-tissue component (arrow). **(G)** Fat-suppressed sagittal contrast-enhanced T1WI delineated irregular enhancement, cortical disruption, and extraosseous extension. **(H)** PET/CT demonstrated markedly increased heterogeneous radiotracer uptake within the distal femoral lesion and its associated soft-tissue component. **(I)** Follow-up CT after surgery showed satisfactory prosthetic reconstruction.

The patient underwent a preoperative CT-guided core needle biopsy. Histopathological examination demonstrated extensive coagulative necrosis without identifiable viable cellular components or definitive evidence of tumor, suggesting that representative tumor tissue had not been obtained (Figure 4A). After written informed consent was obtained from the patient and his family, he underwent wide excision of the distal femoral lesion, total knee arthroplasty, and exploration of the common peroneal nerve. Histopathological examination revealed malignant epithelioid endothelial cells forming irregular vasoformative channels, although cytological atypia was not prominent (Figures 4B,C). IHC showed positivity for CD31, and ERG (Figures 4D,E). The Ki-67 proliferation index was approximately 40% (Figure 4F), supporting the diagnosis of primary EA of the femur. The patient subsequently received 7–8 cycles of postoperative chemotherapy with docetaxel and epirubicin. Follow-up CT performed three months after surgery demonstrated satisfactory prosthetic reconstruction, without imaging evidence of local recurrence (Figure 3I).

**Figure 4:**
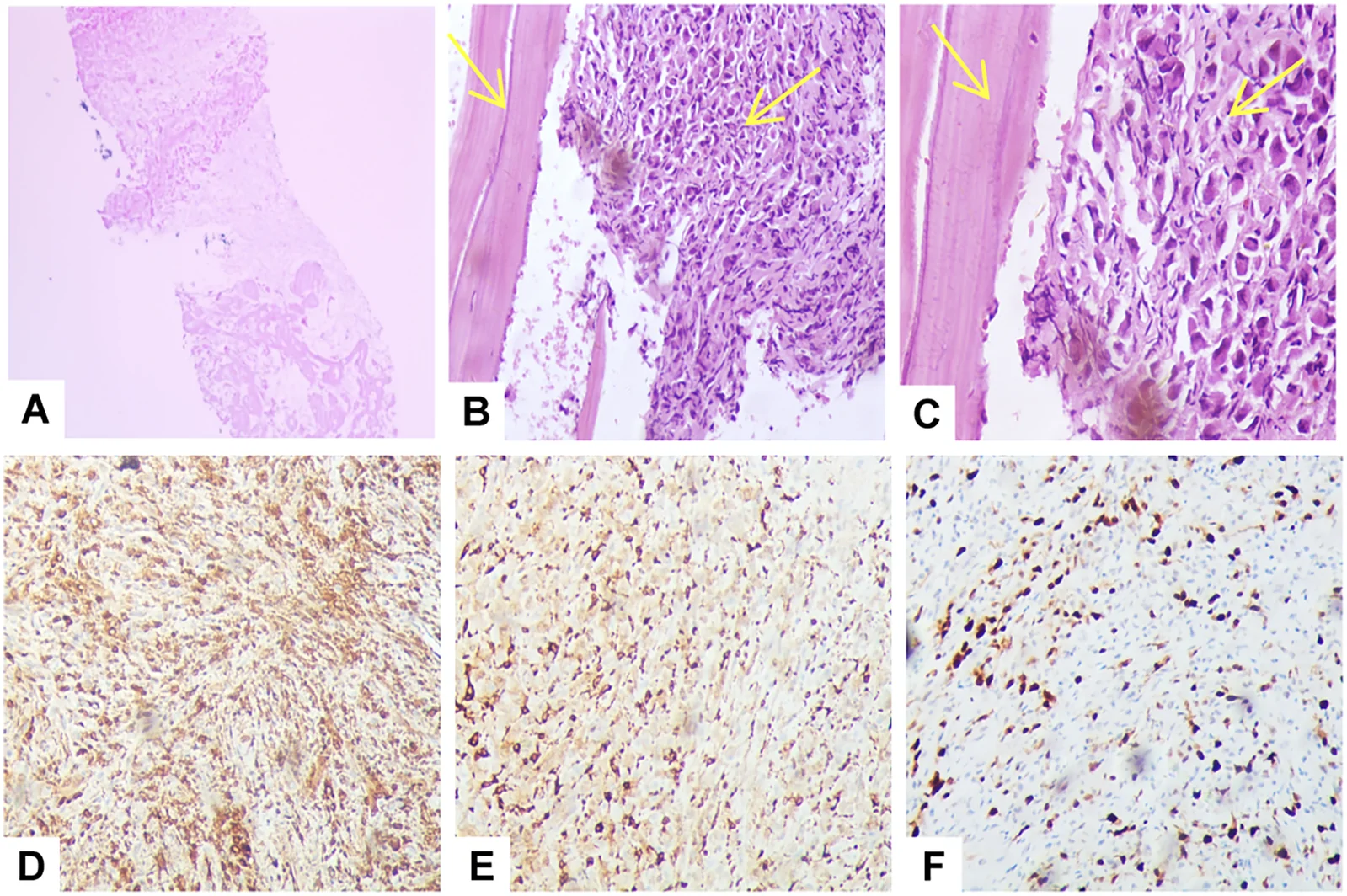
Histopathological and IHC findings of the left distal femoral lesion. **(A)** Preoperative CT-guided core needle biopsy specimen demonstrated extensive coagulative necrosis without identifiable viable cellular components or definitive evidence of tumor. **(B,C)** Low- and high-magnification HE staining demonstrated malignant epithelioid tumor cells forming irregular vasoformative channels, with only mild cytological atypia. The left arrow indicates bone tissue, and the right arrow indicates tumor tissue. **(D)** CD31 was positive in tumor cell. **(E)** ERG was positive in tumor cell. **(F)** The Ki-67 index was approximately 40%.

## Discussion

Angiosarcoma is a rare malignant neoplasm arising from vascular or lymphatic endothelial cells and accounts for approximately 1%–2% of all soft-tissue sarcomas (1, 7). EA is an uncommon, high-grade histological subtype characterized by predominantly malignant epithelioid endothelial cells and is associated with aggressive local invasion, early metastatic dissemination, and an unfavorable prognosis (3, 7). Notably, primary angiosarcoma of bone is exceptionally uncommon, accounting for less than 1% of primary bone sarcomas (2, 8). Herein, we report two rare cases of primary femoral EA in male patients. Preoperative imaging initially suggested plasmacytoma and malignant bone tumor; however, the definitive diagnoses were established by postoperative histopathological and IHC examination. These cases highlight the nonspecific clinical and imaging features of primary osseous EA and underscore the importance of integrating radiologic, histopathologic, and IHC findings to achieve an accurate diagnosis.

The imaging manifestations of malignant vascular tumors of bone are variable and usually lack disease-specific features (5, 9). Radiography and CT typically demonstrate geographic or infiltrative osteolytic destruction, often accompanied by cortical thinning or disruption, endosteal scalloping, and variable extraosseous soft-tissue extension (5, 6, 9). Periosteal reaction is absent in many cases despite the aggressive biological behavior of these tumors. Multifocal skeletal involvement has been reported in approximately 40% of patients with vascular bone tumors and may provide a clue to the vascular origin of multiple osteolytic lesions (9). Furthermore, MRI is useful for delineating intramedullary tumor extent, cortical destruction, hemorrhage, necrosis, peritumoral edema, and adjacent soft-tissue involvement (9). Nevertheless, these findings overlap substantially with those of metastatic carcinoma, plasma-cell neoplasms, giant cell tumor of bone, osteosarcoma, and other aggressive skeletal tumors (5, 9). Consequently, imaging may suggest a malignant bone tumor and aid biopsy targeting and surgical planning, but it generally cannot confirm the endothelial differentiation of the lesion (5, 6, 9).

Metastatic carcinoma and plasma-cell neoplasms should be considered when one or more destructive osteolytic lesions are identified, particularly in the presence of cortical destruction and an extraosseous soft-tissue component (5). Giant cell tumor of bone may also present as an expansile osteolytic lesion; however, it typically arises eccentrically in the meta-epiphyseal region and extends toward the subchondral bone or articular surface (10). Osteosarcoma is more strongly favored when an osteoid matrix or aggressive periosteal reaction is present, although predominantly osteolytic variants may remain difficult to distinguish from malignant vascular tumors (11). Multifocality may support a vascular neoplasm, but it is not pathognomonic because metastatic carcinoma and multiple myeloma can produce a similar distribution (5, 9). Histopathological evaluation is therefore essential when imaging reveals an aggressive osteolytic lesion lacking a characteristic tumor matrix and no primary malignancy has been identified (5, 9). In the present cases, radiography and CT demonstrated expansile osteolytic destruction with cortical disruption and variable extraosseous soft-tissue extension. MRI further revealed heterogeneous intramedullary signal intensity, internal hemorrhagic or hemosiderin-related components, and relatively mild heterogeneous enhancement despite the extensive tumor burden. PET/CT confirmed metabolically active lesions confined predominantly to the affected femur. Although these findings indicated an aggressive malignant bone tumor, they were not specific for EA, emphasizing the need for histopathological and IHC confirmation.

Histologically, osseous EA is composed predominantly of large malignant epithelioid endothelial cells arranged in infiltrative sheets, solid nests, and irregular anastomosing or cystically dilated vascular spaces (3, 12). Tumor cells typically contain abundant eosinophilic cytoplasm and enlarged atypical nuclei with prominent nucleoli and frequent mitotic figures (3, 12). In poorly differentiated areas, recognizable vascular channels may be inconspicuous, and extensive hemorrhage may further obscure the underlying vasoformative architecture (6). These features may lead to misdiagnosis as metastatic carcinoma or another epithelioid malignancy, particularly when the diagnosis is based on a limited biopsy specimen (3, 12). Additionally, osseous EA commonly expresses cytokeratins and endothelial markers (3, 12). CD31 is among the most reliable markers of endothelial differentiation in EA, whereas nuclear ERG expression provides sensitive additional evidence of vascular lineage (12, 13). In a large IHC study, ERG expression was detected in 96 of 100 angiosarcomas (13). By contrast, CD34 expression is less consistent and may be absent in a subset of osseous angiosarcomas (12). Accordingly, a definitive diagnosis should be established through the integrated assessment of histomorphology and a panel of endothelial markers.

The principal pathological differential diagnoses include metastatic carcinoma, epithelioid hemangioendothelioma, and epithelioid sarcoma (3, 12). Metastatic carcinoma may express broad-spectrum cytokeratins but generally lacks convincing vasoformative differentiation and coexpression of specific endothelial markers such as CD31 and ERG (3, 12, 13). Epithelioid hemangioendothelioma usually demonstrates less pronounced cytological atypia, epithelioid cells arranged in cords or nests within a myxohyaline stroma, and most cases demonstrate nuclear CAMTA1 expression (12, 14). Epithelioid sarcoma commonly expresses cytokeratins and may express CD34 but typically shows loss of SMARCB1/INI1 expression (15). In the present cases, coexpression of CK(AE1/AE3), CD31, and ERG confirmed endothelial differentiation despite the epithelial immunophenotype. CD34 expression was absent in one case but present in another, highlighting the immunophenotypic heterogeneity of EA and underscoring the importance of integrated histopathological and IHC evaluation (3, 12).

Complete surgical resection remains the principal treatment for localized primary angiosarcoma of bone (2, 8). In the European Musculoskeletal Oncology Society series, the overall 5-year survival rate was 27%; however, survival differed markedly according to disease stage, reaching 41% in patients with localized disease but only 8% in those presenting with metastatic disease (2). Among patients with localized tumors, the 5-year overall survival rate was 45% in those who achieved complete remission after surgery, compared with 17% in those who did not (2, 16). The role of systemic chemotherapy in primary osseous EA remains unclear, and retrospective studies have demonstrated considerable heterogeneity in treatment regimens. In a retrospective analysis of 60 patients, localized disease was predominantly treated with osteosarcoma-like regimens comprising doxorubicin, cisplatin, and ifosfamide, with or without methotrexate, while paclitaxel was also used (8). Similarly, the European Musculoskeletal Oncology Society (EMOS) series included osteosarcoma-like chemotherapy, doxorubicin with or without ifosfamide, paclitaxel, and gemcitabine (2). Among patients with metastatic disease, partial responses were observed with paclitaxel, whereas stable disease was achieved in some patients treated with gemcitabine, doxorubicin plus ifosfamide, or osteosarcoma-like regimens (2, 17). Collectively, these findings suggest potential activity of taxane- and anthracycline-based chemotherapy; however, no optimal systemic regimen has been established for primary osseous angiosarcoma (2, 8, 18). Weekly paclitaxel also demonstrated antitumor activity in the ANGIOTAX phase II trial, although its specific efficacy in primary osseous EA remains unclear (18, 19). In the present study, the patient in Case 2 received 7–8 cycles of postoperative chemotherapy with docetaxel and epirubicin, with no imaging evidence of local recurrence at the 3-month follow-up. Evidence supporting neoadjuvant chemotherapy in primary osseous EA remains limited, and no standard treatment regimen has been established. Retrospective multicenter studies suggest that neoadjuvant chemotherapy may offer clinical benefit in selected patients with high-risk localized disease, particularly those with larger tumors or a higher predicted risk of death; however, these findings require prospective validation (20, 21). In one multicenter study of angiosarcoma, the most commonly used neoadjuvant regimens were paclitaxel, doxorubicin plus ifosfamide, and gemcitabine plus docetaxel, with a median of six treatment cycles (21). Another retrospective study of localized cutaneous or soft-tissue angiosarcoma reported a pathological complete response in approximately 30% of patients and an R0 resection rate of 80% following neoadjuvant paclitaxel or gemcitabine plus docetaxel, although no significant survival advantage over surgery alone was observed (22). In Case 1, the preoperative CT-guided biopsy was nondiagnostic, and malignancy was not histologically confirmed before surgery. Given the painful pathologic fracture requiring prompt local management, surgical resection was prioritized over neoadjuvant chemotherapy. In Case 2, imaging demonstrated cortical destruction and extraosseous soft-tissue extension without evidence of distant metastasis. Given the extensive osseous involvement and high risk of pathologic fracture, surgical resection was selected as the initial treatment for local disease control. Accordingly, neither patient received neoadjuvant chemotherapy. Radiotherapy may provide additional local control in patients with close or positive margins or other unfavorable pathological features, but its effect on overall survival remains unclear (2, 23). Both patients in the present report underwent wide resection of the femoral tumors followed by endoprosthetic reconstruction, in accordance with the recommended management of localized, resectable osseous EA (2, 23). Given the propensity of osseous EA for local recurrence and early metastasis, long-term surveillance of the surgical site and distant metastatic sites, particularly the lungs and skeleton, is essential (2, 8, 16). Furthermore, future studies are required to know the chemotherapy and targeted agents role in angiosarcoma bone.

In conclusion, we report two rare cases of primary EA involving the femur, in which multimodal imaging combined with histopathological and IHC evaluation established the final diagnosis. These cases broaden the clinical understanding of primary osseous EA and highlight the importance of integrating radiological, pathological, and IHC findings to reduce misdiagnosis and guide appropriate management.

## Data Availability

The raw data supporting the conclusions of this article will be made available by the authors, without undue reservation.
